# Acid-catalyzed transformation of orange waste into furfural: the effect of pectin degree of esterification

**DOI:** 10.1186/s40643-024-00768-2

**Published:** 2024-05-20

**Authors:** Eva E. Rivera-Cedillo, Marco M. González-Chávez, Brent E. Handy, María F. Quintana-Olivera, Janneth López-Mercado, María-Guadalupe Cárdenas-Galindo

**Affiliations:** 1 CIEP Facultad de Ciencias Químicas, Universidad Autónoma de San Luis Potosí, Av. Dr. Manuel Nava No. 6, San Luis Potosí, SLP 78210 México; 2 Ingeniería en Nanotecnología, Universidad de la Ciénega del Estado de Michoacán de Ocampo, Av. Universidad Sur 3000, Sahuayo de Morelos, Michoacán 59103 México

**Keywords:** Pectin hydrolysis, Decarboxylation, Dehydration, Degree of esterification, Furfural

## Abstract

The transformation of biomasses from agro-industrial waste can significantly impact the production of green chemicals from sustainable resources. Pectin is a biopolymer present in lignocellulosic biomass as Orange Peel Waste (OPW) and has possibilities for making platform compounds such as furfural for sustainable chemistry. In this work, we studied the transformation to furfural of OPW, pectins, and d-galacturonic acid (D-GalA), which is the main component (65 wt%) of pectin. We analyzed pectins with different degrees of esterification (45, 60 and 95 DE) in a one-pot hydrolysis reaction system and studied the differences in depolymerization and dehydration of the carbohydrates. The results show that the production of furfural decreases as the DE value increases. Specifically, low DE values favor the formation of furfural since the decarboxylation reaction is favored over deesterification. Interestingly, the furfural concentration is dependent upon the polysaccharide composition of pentoses and uronic acid. The obtained concentrations of furfural (13 and 14 mmol/L), d-xylose (6.2 and 10 mmol/L), and L-arabinose (2.5 and 2.7 mmol/L) remained the same when the galacturonic acid was fed either as a polymer or a monomer under the same reaction conditions (0.01 M SA, 90 min and 433 K). OPW is proposed as a feedstock in a biorefinery, in which on a per kg OPW dry basis, 90 g of pectin and 15 g of furfural were produced in the most favorable case. We conclude that the co-production of pectin and furfural from OPW is economically feasible.

**Graphical Abstract:**

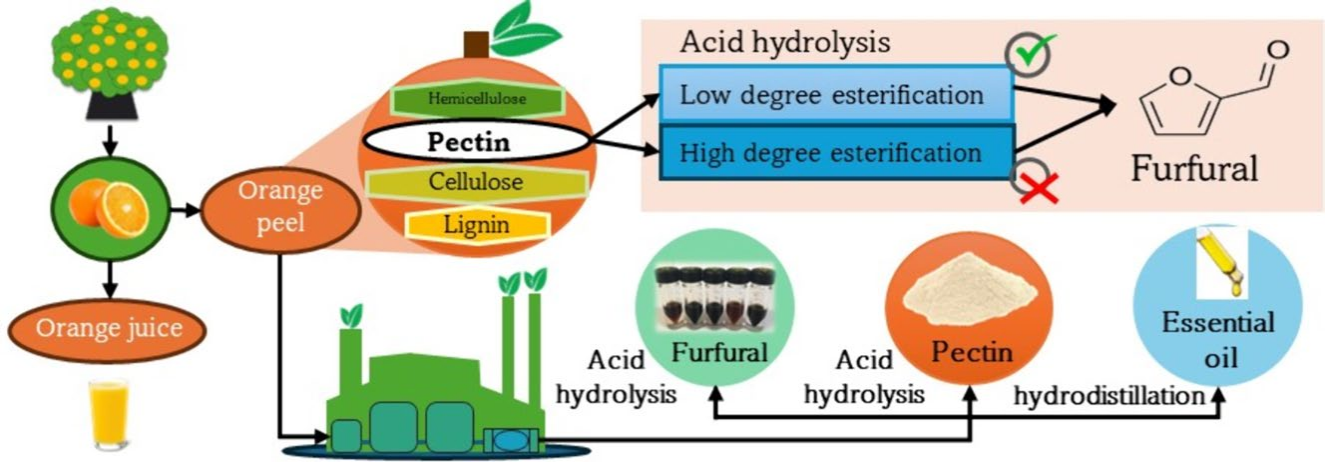

**Supplementary Information:**

The online version contains supplementary material available at 10.1186/s40643-024-00768-2.

## Introduction

The world population has been steadily growing, having attained 8 billion by the end of 2023 (U.S. and World Population Clock 2023). Energy demand, at 1684 PJ/d in 2021 is consequently rising at 0.7% per year and predicted to reach 2056 PJ/d by 2050 (WEO 2023). The use of fossil fuels has accelerated global industrialization and technological development over the years, yet its adverse effects cannot be ignored. Among these effects are rising greenhouse gas emissions responsible for acid rain and climate change, resulting in more floods, melting glaciers, droughts, forest fires, extinction of animal and plant species, and other related issues. The circular bioeconomy has emerged as a novel concept focusing on preventing and remedying the effects of industrialization, with biorefining as one of its main bases. Lignocellulosic biomass is one of the preferred feedstocks for biorefining due to its abundance, availability, and net-zero CO_2_ effect. Pectin-rich biomasses such as citrus waste from orange processing are abundant where citrus crops are used in food processing. In 2018, the United Nations Food and Agriculture Organization (FAO) estimated a citrus production of 104.15 Mt, of which 75.54 Mt corresponded to orange (FAOSTAT 2023). Mexico is amongst the five leading orange producers with a production of 4.74 Mt, which represents 6.3% of the total world production (SIAP 2023). This fruit is used mostly to make juice, jams, and marmalades, processes that generate large amounts of waste (orange peel and pulp).

Orange peel waste (OPW) is a lignocellulosic material rich in pectin (up to 42.7%) (Xiao and Anderson 2013). Unlike hemicellulose, cellulose, and lignin, pectin has been sparsely studied as part of the assessment of feedstocks for biorefining. Pectin is a heteropolysaccharide with a complex structure consisting of 65% of d-galacturonic acid (d-GalA) monomer, reducing sugars such as d-xylose (d-Xyl), l-arabinose (l-Ara), rhamnose (Rha), galactose (Gal), glucose (Glc), and fucose (Fuc) (Romann-Benn et al. 2023; Naqash et al. 2017), linked by α-1,4 glycosidic bonds. The homogalacturonan pectin backbone defines the degree of methyl esterification (DE), which corresponds to the d-galacturonic acid methyl ester content reported as a percentage of the total galacturonic acid. Pectin is a valuable product with applications in the food and pharmaceutical industry, where it is classified according to the DE value. As such, pectin is classified as high methoxy pectin (HMP) if the percentage of carboxyl groups substituted by methyl ester is greater than 50%, and as low methoxy pectin (LMP) if DE is lower than 50% (Sayah et al. 2016). In commercial terms, a high DE value confers gelling properties to pectin, which makes HMP a high value compound in the food and pharmaceutical industries. In contrast, LMP pectin does not have commercial value, and residual biomass that leads to it would not be of interest for pectin extraction but could still have potential applications as a biorefinery feedstock.

The most studied methods for the chemical transformation of biomasses to biofuels and high value compounds involve the use of ionic liquids, super-critical water, hydrothermal, thermal, and chemical treatments (Vaithyanathan et al. 2023; Chen et al. 2017; Nanda et al. 2014; McMillan 2016). Acid hydrolysis is one of the most cost-effective and adequate methods to process residual biomass. It is performed in homogeneous media under mildly acidic conditions, which avoid the use of expensive purification processes and equipment corrosion. According to previous reports, cellulose and hemicellulose treated with acid hydrolysis show high depolymerization to pentoses and hexoses followed by dehydration, which in the former case leads to furfural (Fur), and in the latter to hydroxymethylfurfural (HMF) and levulinic acid. The acids used were methanoic acid, ethanoic acid, HCl, H_2_SO_4_, HNO_3_, or H_3_PO_4_ (Zhou et al. 2015; López et al 2014; Oriez et al. 2019; Świątek et al. 2020; Tao et al. 2024). Furfural has potential for the sustainable production of C5 chemicals via sequential steps of selective hydrogenation and hydrogenolysis. Alternatively, if treated through selective oxidation, it can lead to C4 chemicals (Li et al. 2016). Therefore, furfural could replace crude-oil-based organics to produce resins, lubricants, adhesives, and plastics, as well as valuable chemicals, such as furfuryl alcohol and tetrahydrofurfuryl alcohol.

The hydrolysis of d-GalA, the main component of pectin, has been hardly studied. Furfural has been reported as the main product of d-GalA degradation (Conrad 1931) and the reaction route reported starts with the decarboxylation of d-GalA, followed by a series of dehydration reactions to furfural that involve an intermediary pentose (4,5-unsaturated 4-deoxy-l-arabinose) similar to l-arabinose (Bornik and Kroh 2013). Additionally, resinification and condensation reactions of furfural, involving sugar loss towards unwanted humins, have also been reported (Shi et al. 2020).

The potential of orange peel waste (OPW) as a source of valuable compounds has caught the attention of researchers. Several studies focus on the production of essential oil, flavonoids, pectin, biogas, maleic acid, citric acid, levulinic acid, and biofuels to mention some (Sabater et al. 2022; Ortiz-Sanchez et al. 2024), but furfural is not one of them. These studies focus only on obtaining the compound of interest, and do not consider the integral use of OPW, and typically a significant amount of residue is generated. For other pectin rich biomasses, such as apple, their integral use has been considered by including the fermentation of the residue following pectin extraction (Sabater et al. 2022), but not the possibility of an acid hydrolysis step. The hydrolysis either of the whole OPW or only the residue could warrant the integral use of all the biomass, and several processes within the refinery concept could be developed. For instance, if pectin is extracted first, the amount generated could be too large for the market needs of this product or may not have the desired quality because of its DE. Consequently, the production or co-production of furfural could be an attractive alternative.

The aim of this work is to study the contribution of pectin to the production of furfural from OPW and the influence of the DE in OPW pectin on the yield to this compound. Also, the suitability of using OPW as a feedstock in a biorefinery is evaluated in view of three different processes where furfural is the main product or one of several valuable products.

## Results

### Furfural from polygalacturonic acid

The evaluation of OPW as a feedstock in the biorefinery concept requires an understanding of the transformation of D-GalA, the pectin main component, to furfural, identifying other products formed during the dehydration reaction. At issue here is gaining insight into what other reaction steps may be also present when the pectin depolymerizes.

#### Furfural from d-GalA

**Fig. 1:**
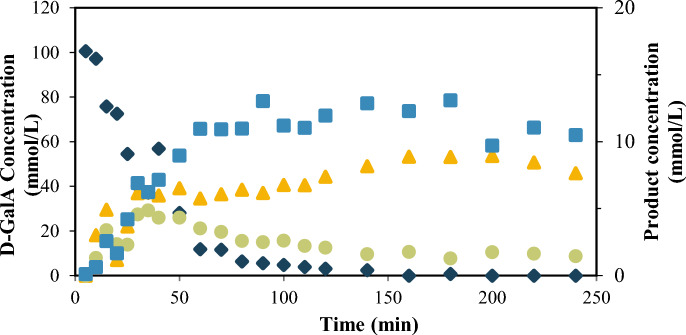
Concentration of D-GalA (diamond) in main axis and its dehydration products D-Xyl (triangle), L-Ara (circle) and Fur (square) in secondary axis. Reaction conditions: T 433 K, catalyst concentration SA 0.01 M.${C}_{oD-GalA}$: 110 mmol/L

Figure 1 shows results for the galacturonic acid dehydration at 433 K in dilute H_2_SO_4_ (SA) solution. The d-GalA protodecarboxylation leads to the formation of L-arabinose as other researchers report (Bornik and Kroh 2013), but also to d-xylose. The dehydration of both sugars is responsible for the presence of furfural (Conrad 1931). According to Garrett and Dvorchik (1969) the steric position of the hydroxyl groups in each sugar favors the dehydration of d-xylose over l-arabinose resulting in higher yields to furfural from d-xylose. The reactivity order in pentoses follows the sequence ribose > xylose ~ lyxose > arabinose with ratios to furfural of 5.4:2.2:2.2:1 under mild acid conditions. The same researchers found that furfural is produced from D-GalA via two reaction pathways: the first has l-arabinose as an intermediate, and the second d-xylose, that directly produces furfural, or after isomerization to l-arabinose. The results in Fig. 1 show that the furfural yield from galacturonic acid after 60 min of reaction is 14%, higher than the yields reported under the same reaction conditions from L-arabinose (5%) or the corresponding d-xylose isomer (10%) (Alonso et al. 2013). The yield was calculated with Eq. (1), where ${n}_{Fur}$ is the number of moles of furfural produced and ${n}_{oD-GalA}$ is the number of moles of galacturonic acid loaded into the reactor.

1 $${yield}_{Fur}=\frac{{n}_{Fur}}{{n}_{oD-GalA}}\times 100$$

**Fig. 2:**
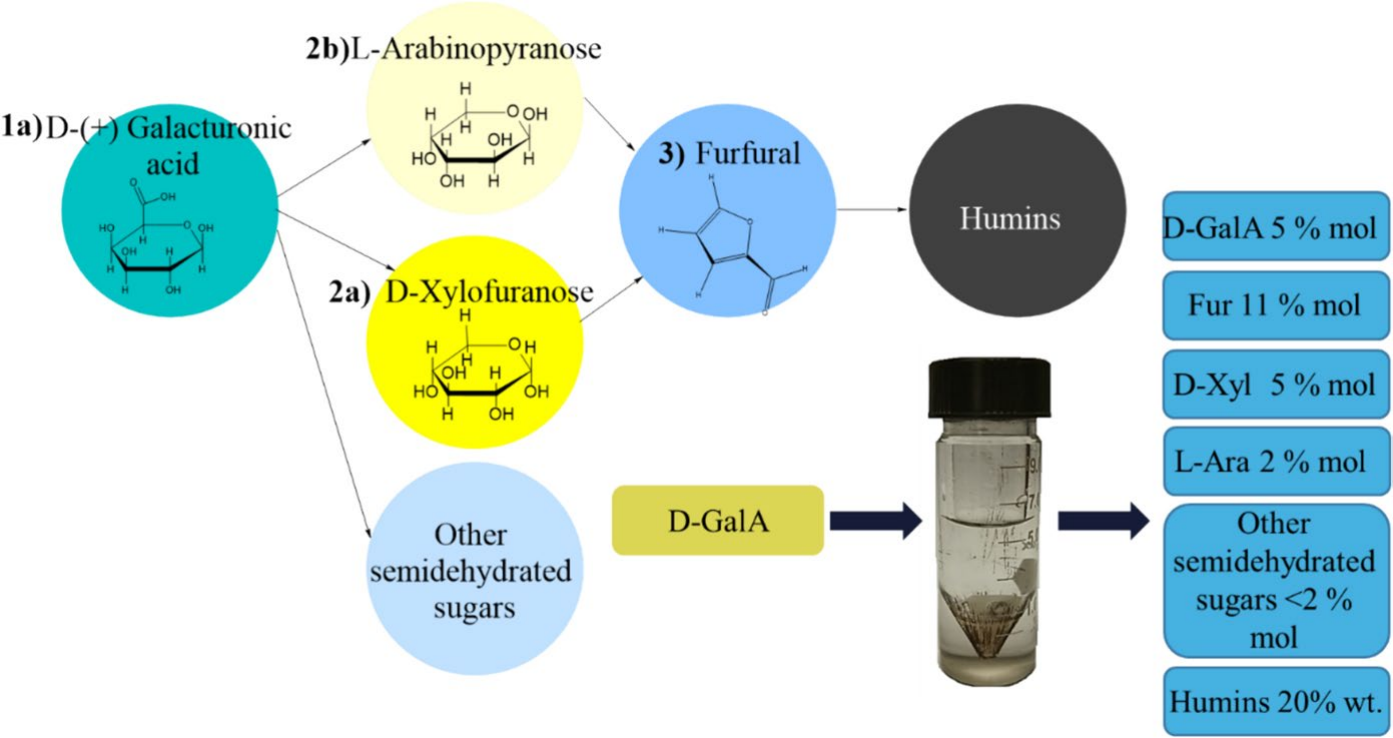
Reaction pathway to furfural formation from d-GalA and product yields after 90 min of reaction, using 0.01 M H_2_SO_4_ as catalyst in aqueous media, at 433 K

After 90 min, more than 94% of d-GalA was consumed. Close to 20 wt% of the d-GalA reaction products correspond to furfural, d-xylose, and l-arabinose, while 22 wt% converted to humins (Fig. 2). Considerably smaller amounts of succinic acid, levulinic acid, glucuronic acid, glucopyranouronic, isomers of galacturonic acid, and galactaric acid were detected via non-quantitative GC/MS analysis (not shown).

#### Polygalacturonic acid depolymerization

**Fig. 3:**
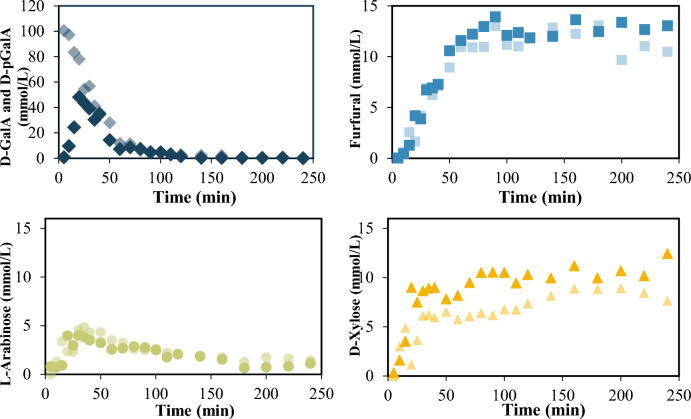
Comparative development of the concentration of the degradation products of d-galacturonic acid in monomer (Blue) and polymer (Black) structure d-pGalA. Reaction conditions:${{\text{C}}}_{{\text{oD}}-{\text{GalA}}}$:110 mmol/L, T 433 K, catalyst SA at 0.01 M.${{\text{C}}}_{{\text{oD}}-{\text{pGalA}}}$:110 mmol/L equivalent of d-GalA

Polygalacturonic acid was used as a model of the homogalacturan in the pectin backbone to study the influence of the depolymerization on the products distribution. Figure 3 compares the products formed from d-GalA and polygalacturonic acid (d-pGalA). The hydrolysis of the polygalacturonic acid, via α-1-4 glycosidic bond cleavage, results in complete depolymerization within 20 min of reaction time.

This process is similar to the depolymerization of other neutral polysaccharides, such as cellulose to glucose (Shrotri et al. 2018) or xylan to d-xylose (Oriez et al. 2019), and is unaffected by the carboxylic groups in d-pGalA. After 20 min of reaction time the furfural, d-xylose, and l-arabinose concentrations are essentially the same, regardless of using the polymer or the monomer as starting material (see Fig. 3). This suggests that depolymerization occurs at a faster rate than decarboxylation. Consequently, the concentration of d-GalA reaches a maximum. Accordingly, the concentration of furfural is the same for both starting materials.

### Pectin depolymerization

To study the effect of the degree of esterification (DE) on the depolymerization and formation of furfural, the hydrolysis products of pectins with three different DE values were studied under the same reaction conditions used for d-pGalA and d-GalA.

#### DE effect on pectin depolymerization

**Fig. 4:**
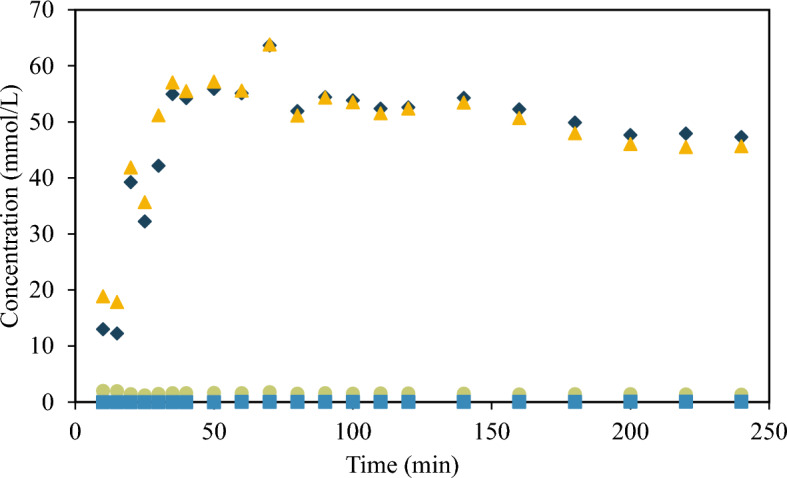
Hydrolysis products of P-95: d-GalA +  d-GalAE (diamond), Fur (square), D-Xyl (triangle) and L-Ara (circle). Reaction conditions: C_oD-GalA + D-GalAE_: 58 mmol/L, C_oNeutral sugars_: 62 mmol*/L, T 433 K, catalyst SA at 0.01 M. * mmol equivalent of pentoses

The concentrations of hydrolysis products derived from the depolymerization of P-95 are shown in Fig. 4. Esterified d-galacturonic acid (d-GalAE) is the main depolymerization product, in agreement with the DE of 95% determined for this material (Table S1). At 40 min reaction time, all the pectin has already depolymerized into d-GalA, d-GalAE, xylose, and a small amount of arabinose. The uronic compounds (d-GalA, d-GalAE) originate from the pectin backbone and arabinose and xylose from the side branches of the P-95. The concentration profiles of uronic compounds and xylose overlap because their concentrations in P-95 are very similar (Table S1), confirming that glycosidic bonds rupture at the same rate independently of monomer type. The formation of furfural is negligible due to the low concentration of d-GalA in P-95, which confirms that it is not produced from d-GalAE. The conversions of xylose and uronic compounds to other products are slow and occur at the same rate, as evidenced by the parallel change in concentrations.

**Fig. 5:**
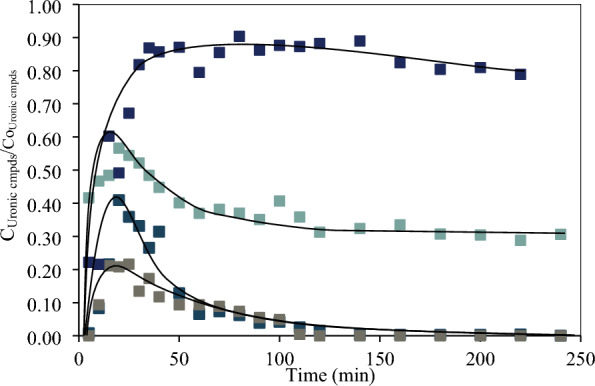
Normalized concentrations of uronic compounds in the hydrolysis of d-pGalA (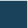), P-45 (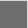), P-60 (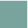) and P-95 (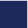). Reaction conditions: P-45: C_oD-GalA + D-GalAE_: 17 mmol/L, P-60: C_oD-GalA + D-GalAE_: 38 mmol/L and P-95: C_oD-GalA + D-GalAE_: 58 mmol/L T 433 K, catalyst SA at 0.01 M

Pectins with lower esterification degrees (45% and 60%) were also tested. The maximum concentration (d-GalA +  d-GalAE) obtained from P-60 occurs at 20 min (see Fig. 5) and is very close to the content of these monomers in the pectin. For P-60 the rate of conversion to other products is faster than for P-95, and results from a higher content of d-GalA, confirming that the esterified d-GalAE is not converted easily to other products. P-45 is fully depolymerized also at 20 min reaction time, and the higher proportion of d-GalA in this pectin results in a maximum uronic compounds concentration below the content of d-GalA and d-GalE in the pectin. This is similar to the p-GalA depolymerization. Unlike the other two pectins, both d-GalA and d-GalAE in P-45 completely convert to other compounds, and the reaction terminates at 110 min.

Although the reaction conditions used for the pectins evaluation were the same as used for d-pGalA, the difference in reaction rates and conversion are related to changes in the reactive mixture properties during the reaction. One important factor is the influence of the pH, and this is discussed in another section.

#### Decarboxylation: xylose and arabinose concentration

**Fig. 6:**
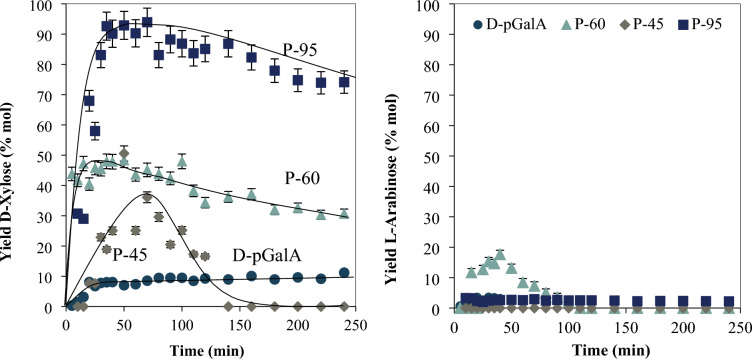
Yield to d-xylose and l-Arabinose for each pectin tested. Reaction conditions: T 433 K, catalyst SA at 0.01 M

Pectin side branches may contain l-arabinose and d-xylose that are liberated during depolymerization. The aforementioned sugars may also result directly from the protodecarboxylation of the galacturonic acid produced during depolymerization. Figure 6 shows the yields to these saccharides calculated according to Eq. 2,

2 $${yield}_{NS}=\frac{{n}_{NS}}{{n}_{o Xyl}+{n}_{o Ara}+{n}_{oD-GalA}}$$

where: ${n}_{NS}$ = xylose or arabinose concentration. ${{\text{n}}}_{\mathrm{o D}-{\text{Xyl}}}$= xylose monomer content in pectin. ${{\text{n}}}_{\mathrm{o L}-{\text{Ara}}}$= arabinose monomer content in pectin. ${{\text{n}}}_{\mathrm{o D}-{\text{GalA}}}=$ d-GalA monomer concentration in pectin.

For all the pectins, the yield to d-xylose shows a maximum that evidences its further conversion to other compounds, the only exception is d-pGalA, which shows a continuous increment, quick at first and that slows down after 20 min.

Compared to P-95 and P-60, d-xylose conversion to other compounds is faster for P-45 and at 140 min reaction time there is not xylose left with this pectin. Differences in reaction rates can be attributed to changes in the pH. This effect will be discussed in the following section.

From P-60 the yield to l-arabinose is higher than from the other pectins, that cannot be attributed to d-GalA conversion to arabinose, but to a higher content of arabinose in the pectin.

### Furfural from pectin with different DE

#### Furfural yield and DE

**Fig. 7:**
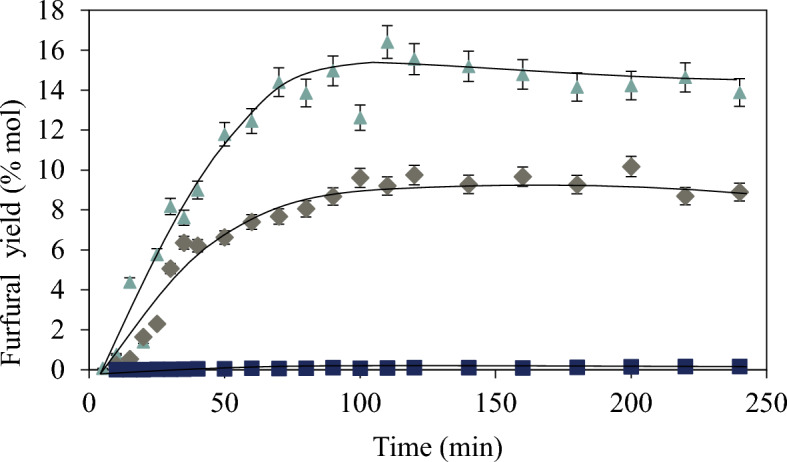
Furfural yield for each pectin tested. P-95 (square), P-60 (triangle), P-45 (diamond) at T 433 K and pH = 2, with SA as catalyst

There is not appreciable furfural yield from P-95. As shown in Fig. 7, furfural yield varies with DE, but not in a systematic way. The pH changes slightly during the reaction (± 0.1), with exception to P-45 which shows an increase of 0.6 units (see Table S2). It is also noteworthy that the initial pH for the P-45 reaction mixture is 3.9, which then increases to a final value of 4.5, while for the other two is between 2.1 and 2.6.

At higher pH the selectivity to norfuraneol (not shown here) is favored. Other authors (Bornik and Kroh 2013) report similar results at high pH values.

#### Effect of initial pH on furfural yield

**Fig. 8:**
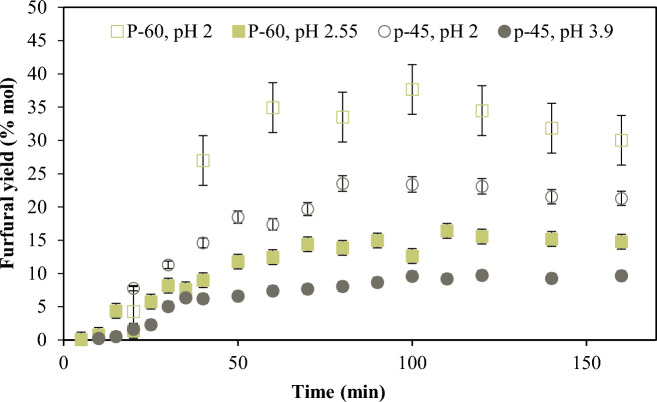
Furfural yield from hydrolysis of P-45 (circle, open circle) and P-60 (square, open square) with 0.05 M SA and with 0.01 M SA. Reaction conditions: C_oD-GalA + D-GalAE_: 17 mmol/L, C_oNeutral sugars_: 119 mmol/L as equivalent pentoses, T 433 K. P-45: SA 0.01, M pH(i): 3.9 and SA 0.05 M, pH(i) 2, and P-60: SA 0.01 M, pH(i) 2.55 and SA 0.05 M, pH(i) 2

If the reaction is performed with a higher concentration of sulfuric acid to adjust the initial pH to 2, the yield to furfural from P-45 and P-60 increases in both cases as Fig. 8 shows. The increment is more important with P-60. The formation of furfural shows a decrement at high reaction times suggesting that it is being degraded into other compounds, for example humins.

#### Humins formation

**Fig. 9:**
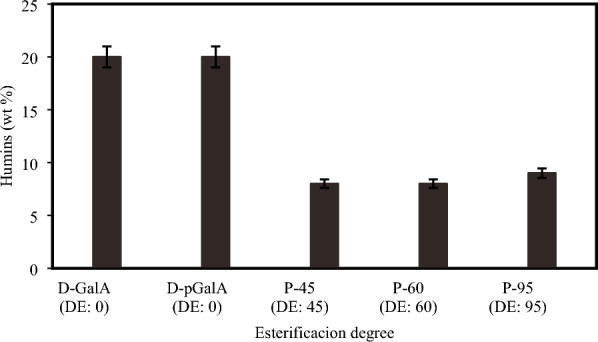
Humins formation from different biopolymers. Reaction conditions: reactant 2 wt. %, 0.01 M SA, 433 K at 90 min of reaction

Humins are dark colored solids formed during sugars dehydration. In this work, humins were quantified at reaction time of 90 min. A higher percentage of humins were obtained from d-GalA and d-pGalA than from P-45, P-60 and P-90 (see Fig. 9). At reaction conditions similar to those used in this work, humins formation from neutral sugars as d-xylose, l-arabinose and glucose is associated with resinification reactions of products such as furfural, levulinic acid and HMF, among others (Liu et al. 2022; Constant et al. 2024; Patil et al. 2012; Shi et al. 2020). This could explain humins formation from P-45 and P-60, but not from P-95 which does not yield furfural, yet all three pectins produce similar humin yields. There should be other sources of humins. Levulinic acid and formic acid were also detected in this work, and according with the literature these compounds are also precursors of humins (Tsilomelekis et al. 2016).

#### Furfural yield from OPW pectin

The formation of furfural from OPW will be favored if the content of pectin is high in the orange peel waste, but the yield also depends on the presence of other biomass components capable of producing furfural, such as hemicellulose. To analyze the transformation of OPW to furfural with hydrolysis three cases were considered: the first involves the use of dried orange peel biomass (OPW); in the second, the pectin was first extracted from OPW in a HCl solution at pH 2.9 at 358 K for 90 min; and in the third, the pectin was extracted using optimized extraction conditions previously reported in the literature (1 wt% H_2_SO_4_ for 60 min at 368 K)(Vaez et al. 2021). The pectins extracted in the last two cases have respectively, DE of 55 and 60 and homogalacturonan content of 65 and 70 wt%, meeting minimum requirements (65%) of commercial pectins (Müller-Maatsch et al. 2016)**.** However, the yield to pectin was almost triple with the use of SA (25% vs 9%) (Table S3). This difference is consistent with previously reported results indicating that acid type and concentration for the hydrolysis are important factors (Rungraeng and Kraithong 2020). Higher yields were reported with SA vs HCl (Arrollado et al. 2018).

During pectin extraction from OPW, other biomass components (hemicellulose and cellulose) undergo depolymerization, but the subsequent sugar degradation processes are very slow or non-existent (Anukam and Berghel 2021). Therefore, processes based on the second and third cases could produce pectin as a by-product and furfural from dehydration of the remaining sugars. The residual solids in case 3 (25 wt% of dry OPW) were lower than in case 2 (55 wt%), and they are composed of hemicellulose, cellulose, and lignin. In case 2 the depolymerization of hemicellulose and cellulose during pectin extraction occurred to a lower degree than in case 3 due to the lower acid concentration, which yielded lower dissolved sugar content (36 wt% vs 50 wt%).

**Fig. 10:**
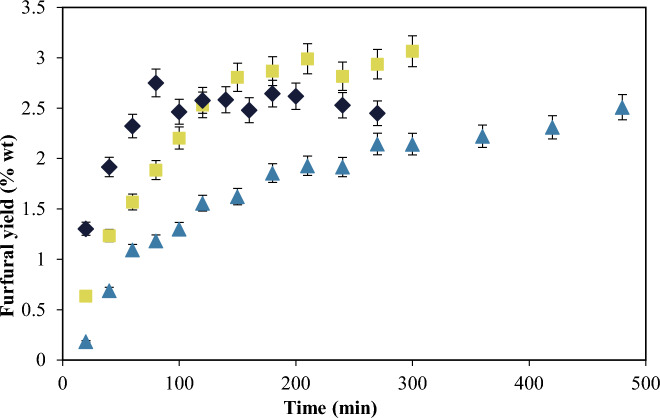
Furfural production from OPW with and without pectin at 433 K using sulfuric acid 0.05 M as catalyst and initial biomass concentration of 10% w/w. OPW (triangle), BWPLE (square), BWPSE (diamond)

Without the pretreatment the formation of furfural is slower as shown in Fig. 10, and continues to form after 500 min, while from the solids without pectin produced in case 2 (BWPLE) and case 3 (BWPSE) the maximum furfural yield already occurs at 150 min. The pre-hydrolysis made more accessible the polysaccharides to depolymerization and dehydration to produce furfural.

### Orange peel waste as a biorefinery feedstock

Pectin is a valuable product with different applications and its extraction for commercial purposes should be considered by any process based on the use of the orange peel waste. The results reported in the previous sections were used to develop three different processes within the biorefinery concept: Process 1 is based on OPW used only to produce furfural as main product; Process 2 considers pectin extraction with HCl and furfural production from BWPLE; Process 3 has as products pectin and furfural from BWPSE.

**Fig. 11:**
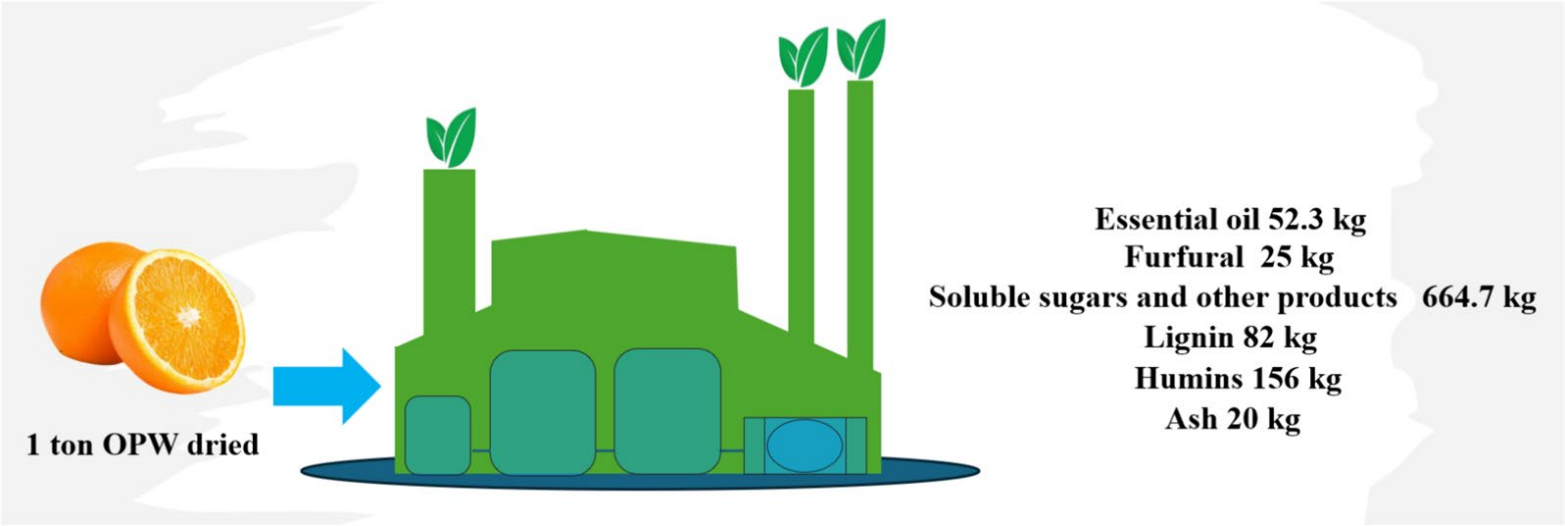
Products yield from OPW without pectin extraction (Process 1)

Pectin and furfural yields obtained in this work were used to evaluate the economic potential of the processes based on the products value; the feedstock value was assumed zero since it is a waste biomass. The results are shown in Table 1. The economic potential was also calculated including the production of essential oil. The yield to this product (52.3 kg/ton) is based on previous reports for its extraction from OPW (Ortiz-Sanchez et al. 2021), which at a price of 10 $/kg represents an important contribution to the products value. The first step of each process would be the essential oil extraction. The mass balance results for Process 1 products are shown in Fig. 11.

**Table 1:** Economic potential of pectin and furfural produced from OPW with and without pectin extraction

Process	Reaction time (min)	Furfural (kg/ton*)	Pectin (kg/ton*)	Total Products Value without Essential Oil ($/ton*)	Total Products Value without Essential Oil ($/ton*)
1	480	25	0	55	630
2	210	14.5	90	1022	1597
3	80	6	250	2763	3339

The reaction time corresponds to the maximum furfural yield. Essential oil yield is 52.3 kg/ton

*Dry basis biomass

Furfural value 2.2 $/kg (Ortiz-Sanchez et al. 2021).

Pectin value 11 $/kg (Ciriminna et al. 2016).

**Fig. 12:**
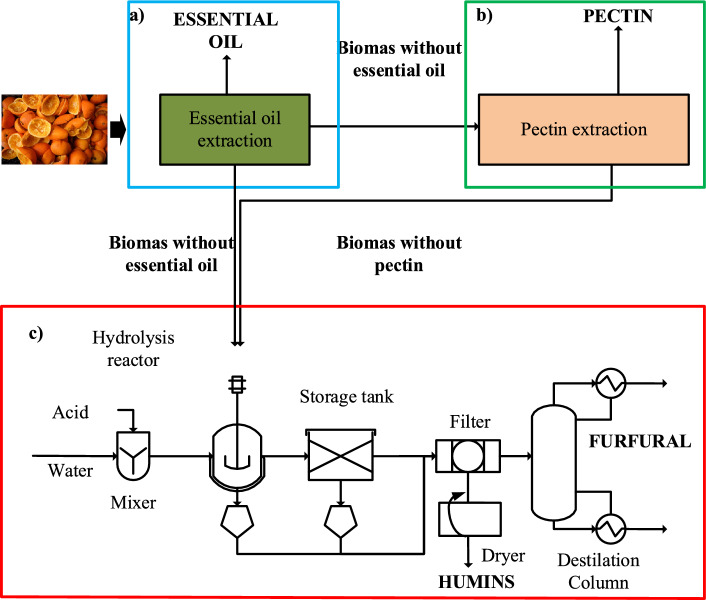
Process flow diagram of the proposed co-production essential oil, pectin and furfural system. **a** Essential oil process block **b** Pectin extraction process block and **c** Furfural production process block

Even though Process 1 yields the largest amount of furfural, Process 3 is the most economically viable because it yields more pectin. While the possibility exists that OPW supply may exceed the market demand for commercial-grade pectin, it would be desirable to process only a portion of OPW sufficient to meet the market demand, leaving the rest of the biomass for direct conversion to furfural. A biorefinery based on this strategy should thus include each of these individual processes (extraction of essential oil, pectin extraction and furfural production), as shown in Fig. 12.

Assuming the cost of raw materials typically corresponds to 60% of the total production costs (Peters et al. 1991), the use of a residual biomass with a zero cost could represent savings of 60%. A detailed economic and life cycle analysis (LCA) would be desirable to evaluate the commercial feasibility and environmental sustainability of the processes analyzed in this work.

OPW use in a biorefinery will produce gas, liquid and solid emissions depending on the processes involved. The integral use of the biomass would have as a major emission factor the production of CO_2_. This gas is responsible for global warming, and it is desirable to reduce or eliminate its emissions. If the boundaries for the LCA are defined as the process boundaries, the emissions of CO_2_ to the environment will be larger than zero. CO_2_ is formed by decarboxylation of D-GalA, necessary to produce furfural. Other biomass components (hemicellulose) that produce furfural would not emit CO_2_ since they only involve dehydration reactions. Therefore, the LCA is restricted to CO_2_ emissions for the processing of 1000 kg of OPW in the 3 cases proposed in this study. Table 2 shows these results. The evidence here shows that CO_2_ emissions decrease when more biomass in converted to pectin and less to furfural (Process 3).

**Table 2:** CO_2_ emissions from furfural production from D-GalA. The CO_2_ emissions from the combustion processes involved in energy generation for the process were not included

Process	CO_2_ (kg/ton OPW)
1	11.5
2	6.6
3	2.7

## Discussion

Several researchers have explored the use of OPW as a raw material in the biorefinery concept to produce valuable products (Vaez et al. 2021; Anukam and Berghel 2021; Ortiz-Sanchez and Cardona-Alzate 2021; Ortiz-Sanchez et al. 2021; Vigneshwar et al. 2022). However, furfural is not among these products. Given the substantial amount of waste generated from orange processing in the food industry, it would be impractical to dedicate the entirety of this waste to the production of a single product.

The furfural yield obtained through OPW hydrolysis is too low to make a process economically viable solely based on its production. However, the consideration of other products, such as pectin and essential oil, increases the economic feasibility of an industrial process based on the use of OPW as we show in this work. Therefore, a biorefinery should consider the integral use of the biomass to produce multiple products.

The pectin yield can be optimized by controlling extraction conditions and its production level is only linked to market demands. Essential oil production is also limited by market demands but it is highly desirable because of its high value. Furfural is not the most expensive product, but the market possibilities could be higher than for the other two because furfural is a platform molecule; therefore, a high production volume would make the process more profitable. But a higher yield to furfural from OPW would be desirable. The sources of this compound in the OPW are pectin and hemicellulose that produce the arabinose, xylose and d-GalA that are converted to furfural. In this work we found that the yield to furfural from d-GalA is similar to that reported from other sugars when water is used as a solvent and H_2_SO_4_ as a catalyst (Lavarack et al. 2002). Other reaction conditions must be identified to increase the yield to make more selective the conversion of d-GalA, arabinose and xylose to furfural, and reduce the formation of humins. There is the possibility that the use of heterogeneous catalysts and other solvents may give results more favorable, as other researchers have found in the formation of humins from glucose and fructose (Liu et al. 2022; Constant et al. 2024).

The conversion of xylose to furfural is very low in this work. It is produced from d-GalA but unlike arabinose its concentration remains unchanged for several hours of reaction time. However, the use of another solvent could increase the conversion, for instance, the use of deep eutectic solvents results in a furfural yield of more than 50% from xylose at 170 °C in 30 min (Liu et al. 2023b; Yang et al. 2023a, b; Xu et al. 2023; Lei et al. 2023); under similar conditions if water is used as a solvent the conversion of xylose is barely perceptible after 4 h of reaction time (this work). Also, the possibility of increasing the furfural yield with a heterogeneous catalyst should be explored. For example, H-Y zeolite-based catalysts yield 77.5% furfural with a xylose conversion close to 100% (Wang et al. 2022). It remains to be seen if similar results could be obtained with pectin derived xylose.

The food industry requires pectin with high DE, therefore pectin rich biomasses that produce pectin with a lower DE would be a more desirable feedstock for furfural production. As we found in this work, the yield to furfural increases as the DE decreases. Even when there is the possibility of converting esterified d-GalA to d-GalA because of the reversibility of this reaction, this process may occur with a low reaction rate since the formation of furfural was not observed with P-95. However, the degree of esterification does not determine entirely the furfural yield, this is really a consequence of the concentration of d-GalA in the biomass.

The possibility of co-production of essential oil, pectin and furfural is not the only possibility as other researchers have found. Within the biorefinery, furfural could be used to obtain other compounds as it is demonstrated in the production of furfurylamine, furfuryl alcohol and furoic acid (Liu et al. 2023a; Lei et al. 2023; Yang et al. 2023b; Xu et al. 2023). Other possibilities open up if the residual biomass is pyrolyzed to produce biochar (Yang, et al. 2023a, b) that could be the base of heterogeneous catalysts that could be used in the hydrolysis process. Even if the conversion to furfural is not high, the high level of saccharides in the remnant solvent would make it suitable for, for example, in the production of alcohols.

## Conclusions

Furfural production from galacturonic acid as a monomer or polymer (d-GalA and d-pGalA), pectins with different DE and OPW with and without pectin were analyzed. According to the results, d-GalA and D-pGalA show no difference in the reaction pathways for the formation of furfural: the yield to furfural, neutral sugars, and humins remained the same in both cases.

Un-esterified galacturonic acid favors furfural production. This is due to the irreversible decarboxylation reaction followed by consecutive dehydrations under mild acid reaction conditions. Furthermore, the methyl ester group present in uronic acid confers reactive stability to the galacturonic acid molecule at this reaction conditions, and therefore, pectin with high content of d-GalA should be preferred as raw material to furfural production in biorefinery processes. Both OPW with and without pectin potentially can compete with other raw materials used as feedstock in furfural production under similar conditions. Additionally, the obtention of other valuable products such as essential oil, pectin and sugars ensures the economic feasibility of a biorefinery based on OPW as a feedstock.

The use of other solvents and heterogeneous catalysts should be explored to increase the yield to furfural.

## Materials and methods

### Chemicals

d-( +)-Galacturonic acid monohydrate, Poly-d-( +)-Galacturonic acid, furfural (Sigma Aldrich, 97%), poly-d-( +)-Galacturonic acid methyl ester (P-95), Low (P-45) and High (P-60) methoxy pectin denominated (Carbsynth, 97%), d-( +)-Xylose (Golden bell, 97%), l-( +)-Arabinose (Jalmek, 97%), ethyl acetate and anhydrous sodium carbonate (ACS, 99.9%, 99.9%), sulfuric acid (Fluka, 96.4%), ethanol (AZ, 96°) and fresh Valencia oranges (Citrus Sinesis) were used as the orange peel source.

### Orange peel waste pretreatment

Fresh Valencia orange, i.e., (Citrus sinensis), cultivated in Rioverde, S.L.P, was used as a feedstock. The fruit was washed, and the juice squeezed. The peel was cut in small pieces to favor homogeneous drying at 333 K for 48 h. The orange peel waste was periodically stirred to ensure homogeneous drying. The dry peel was pulverized and sifted to particle size between 35 and 50 mesh (297 and 500 μm) and stored at room temperature for further use.

### Pectin extraction

Two methods previously reported in the literature were used for the pectin extraction. In one method with 20 wt% of OPW (light conditions), hydrolysis of was done with HCl in water with a pH of 2.9 during 90 min at 358 K (Marín-Proa 2012). The second involved the optimum conditions, namely, with 20 wt% of OPW, 1 wt% of H_2_SO_4_ in the biomass aqueous solution at 368 K during 60 min (Vaez et al. 2021). For both extractions the reaction was stopped in an ice-water bath, followed by solid/liquid separation by centrifugation. In this separation supernatants contain the pectin, and the residue is the biomass without pectin. For the pectin extraction pH was adjusted to 3.5 in agreement with the best conditions reported for pectin separation (Vaez et al. 2021). Pectin was separated after adding alcohol 96° with a relationship of 1:1 v:v to pectin liquor, the gelled solution was stored for 24 h at 277 K and separated by centrifugation for 20 min at 5600 rpm. Pectin was dried for 48 h at 333 K, grinded, sieved to a particle size between 297 and 500 μm and stored for further characterization. The orange peel waste without pectin was also evaluated for the furfural production. The residual biomass from the light hydrolysis (BWPLE) was dried for 48 h at 333 K. The biomass without pectin from the strong extraction conditions (BWPSE) was washed with deionized water to obtain a water a pH of 2 in the washing water. This biomass was dried at 333 K for 48 h.

### Methyl esterification degree

The DE value of all pectin biopolymers was determined by the titrimetric method (Bochek et al. 2001), two titrations were carried out to determine the DE value. The first served to quantify the unesterified carboxyl groups and the second for the de-esterified carboxyl groups, after the saponification. The free esterified carboxyl groups moles were titrated with a solution 0.1 N NaOH (nCOOH). Following, the number of esterified carboxyl groups was saponified by addition of 10 mL of 0.1 N NaOH solution. The previous mix was stirred at room temperature for 2 h to complete saponification of the esterified carboxyl groups of the polymer, followed by addition of 10 mL of 0.1 N HCl. The unesterified carboxyl groups were titrated with the corresponding volume of 0.1 N NaOH. The percentage of the esterified carboxyl groups (nCOOCH_3_) was calculated from the volume of 0.1 NaOH solution spent for titration. The following equation was used to calculate the DE value:

1 $$DE = \frac{{n_{{COOCH_{3} }} }}{{n_{T} }} \times 100$$

2 $${n}_{T}={n}_{{COOCH}_{3}}+{n}_{COOH}$$

3 $${n}_{COOH}={N}_{1NaOH}*{V}_{1NaOH}$$

4 $${n}_{{COOCH}_{3}}={N}_{1NaOH}*{V}_{2NaOH}$$

### Pectin transformation to furfural

The chemical treatment was carried out in a batch reactor with capacity of 10 mL, using sulfuric acid solution (0.01 M) as solvent. The reactants evaluated in this system were d-GalA, d-pGalA, P-45, P-60, and P-95. A concentration of 2% w/w of reactant was used in every case, equivalent to 0.0834 g of the reactant and 4.087 g of the acidic solution. The reaction occurred in sealed vessels under autogenous pressure at 433 K in a glycerin bath. The reaction vessels were open at different periods of time (5, 10, 20 or 30 min). The reactors were maintained in constant agitation at 700 rpm. The reaction was stopped by reactor immersion in an ice-water bath. These reaction conditions are similar to those reported as optimal for furfural production from other sugars (Gallo et al. 2013). The samples were placed in Eppendorf tubes and analyzed by High Performance Liquid Chromatography (HPLC) as soon as possible. Each sample was covered with aluminum to prevent further degradation of the furfural present in the sample. The reaction products were quantified using. The molecular exclusion method was carried out using the Agilent Technologies 1260 Infinity equipment, equipped with UV-DAD (Agilent Technologies) and UV-DAD-RID detectors. The wavelength of the detector was set at 278 nm. An acetonitrile solution (0.6 mL/min flow) was used as mobile phase. A C18 column (Agilent, particle size 5 microns, 150 mm length), maintained at 308 K, was used for the analysis of furfural. An HC-75 Ca^++^ column (Agilent) was used to analyze organic acids, d-xylose, and l-arabinose. The mobile phase was H_2_SO_4_ 0.005 M (0.6 mL/min). Before HPLC injection, the samples were centrifuged for 1 min at 10,000 rpm and corresponding dilutions were made in each case: the dilutions to detect furfural and derivatives was 1:50 and for sugars was 1:3.

### Furfural production from biomass

Furfural was produced from orange peel waste (OPW), and orange-peel waste without pectin (BWPLE and BWPSE). The reaction conditions were the same as used for the pectin transformation to furfural. The concentration of the acidulated solution was 0.05 M H_2_SO_4_ and the biomass-solution relationship was 10% w/w. The reaction mixture was neutralized with sodium carbonate and furfural extraction was performed with 2 volumes of ethyl acetate. The remaining water was removed with anhydrous sodium sulfate. The quantification of furfural present in these samples was carried out using a chromatograph model 6890N Network GC System connected to a selective mass detector model 5973 Network (MSD) (Agilent Technologies, Wilmington, DE, USA). The detection conditions were: injection port temperature 523 K, temperature ramp from 323 to 503 K with a heating rate of 20 K/min, ZB-Wax column (30 m × 0.32 mm 0.50 µm) and N_2_ as carrier gas at 45 mL/min. A calibration curve was used to calculate the furfural concentration.

### Humins quantification

Humins were separated from the reaction products mixture by filtration and dried at 333 K before weight measurement.

All the experiments were run in duplicate, the standard error calculated, and error bars added in the corresponding graphics and tables.

### Life cycle assessment

For purposes of this study, LCA is restricted to CO_2_ emissions. The CO_2_ produced in the biomass transformation was evaluated according to the reaction stoichiometry:

$${C}_{6}{H}_{10}{O}_{7}\rightarrow{C}_{5}{H}_{4}{O}_{2}+{{CO}_{2}}$$

Using the furfural values obtained in each process scenario being studied.

### Supplementary Information

Additional file 1.

## Data Availability

Data may be made available on request.

## Abbreviations

OPW: Orange peel waste
d-GalA: d-Galacturonic acid
d-pGalA: d-polygalacturonic acid
d-GalAE: Esterified d-galacturonic acid
DE: Degree of esterification
D-Xyl: d-Xylose
l-Ara: l-Arabinose
Rha: Rhamnose
Gal: Galactose
Glc: Glucose
Fuc: Fucose
HMP: High methoxy pectin
LMP: Low methoxy pectin
SA: Sulfuric acid
P-95: Pectin with a degree of esterification of 95%
P-60: Pectin with a degree of esterification of 60%
P-45: Pectin with a degree of esterification of 45%
Fur: Furfural
HMF: Hydroxymethylfurfural
BWP: Biomass without pectin
BWPLE: Biomass without pectin with HCl pectin extraction
BWPSE: Biomass without pectin with SA pectin extraction
