# Supplementary material for: Acid-catalyzed transformation of orange waste into furfural: the effect of pectin degree of esterification
Source: Bioresour Bioprocess. 2024 May 20;11(1):52. doi: 10.1186/s40643-024-00768-2 (PMC11106045; doi:10.1186/s40643-024-00768-2)
Supplement: Supplementary file 1 — Additional file 1. [file 40643_2024_768_MOESM1_ESM.docx]

SUPPLEMENTARY INFORMATION

**Acid-catalyzed transformation of orange waste into furfural: the effect of pectin degree of esterification**

Eva E. Rivera-Cedillo^1^, Marco M. González-Chávez^1^, Brent E. Handy^1^, María F. Quintana-Olivera^1^, Janneth López-Mercado^2^ and María-Guadalupe Cárdenas-Galindo^1,^*

^1^ CIEP Facultad de Ciencias Químicas, Universidad Autónoma de San Luis Potosí, Av. Dr. Manuel Nava No. 6, San Luis Potosí, S. L.P., C.P. 78210, México

^2^ Ingeniería en Nanotecnología, Universidad de la Ciénega del Estado de Michoacán de Ocampo, Av. Universidad Sur 3000, Sahuayo de Morelos, Michoacán, C.P. 59103, México.

*corresponding author. EMAIL: [cardenas@uaslp.mx](mailto:cardenas@uaslp.mx)

**Table S1**

Pectin composition.

| **Reactive** | **DE** | **D-GalA**  **(mmol/g Pectin )** | **D-GalAE**  **(mmol/g Pectin )** | **Neutral sugars**  **(mmol*/g Pectin)** |
| --- | --- | --- | --- | --- |
| D-GalA | 0 | 5.15 | 0 | 0 |
| D-pGalA | 0 | 5.15 | 0 | 0 |
| P-45 | 45 | 0.38 | 0.41 | 5.59 |
| P-60 | 60 | 0.7 | 1.06 | 4.29 |
| P-95 | 95 | 0.13 | 2.58 | 2.92 |

**Table S2**

Changes in pH after dissolution of sugars and pectins in deionized water, pH (0); dissolution in 0.01M sulfuric acid solution, pH(i); complete depolymerization (90 min of reaction time), pH(f).

| **Reactant** | **pH (0)** | **pH (i)** | **pH (f)** |
| --- | --- | --- | --- |
| D-Agal | 2.25 | 2.11 | 2.11 |
| D-pAgal | 2.97 | 2.11 | 2.18 |
| P-45 | 4.35 | 3.91 | 4.53 |
| P-60 | 3.1 | 2.55 | 2.78 |
| P-95 | 7 | 2.33 | 2.46 |

**Table S3**

Products obtained from OPW. The weight percentage is calculated in reference to OPW dry basis. Case 2 uses HCl as the catalyst, Case 3 uses SA as the catalyst.

|  | **Case 2**  **(wt%)** | **Case 3**  **(wt%)** |
| --- | --- | --- |
| **Pectin** | 9 | 25 |
| **Solid biomass without pectin (BWP)** | 55 | 25 |
| **Other compounds (Dissolved sugars)** | 36 | 50 |
